# Self-reported mental health status of pregnant women in Sweden during the COVID-19 pandemic: a cross-sectional survey

**DOI:** 10.1186/s12884-022-04553-x

**Published:** 2022-03-28

**Authors:** Chung Ho-Fung, Ewa Andersson, Huang Hsuan-Ying, Ganesh Acharya, Simone Schwank

**Affiliations:** 1 Department of Psychology, The University of Hong Kong, Hong Kong, China; 2 Department of Anthropology, The Chinese University of Hong Kong, Hong Kong, China; 3 Department of Women’s and Children’s Health, Division of Reproductive Health, Karolinska Institutet, Stockholm, Sweden; 4 Division of Obstetrics and Gynecology, Department of Clinical Science Intervention and Technology (CLINTEC), Karolinska Institutet and Center for Fetal Medicine, Stockholm, Sweden; 5 Department of Obstetrics and Gynecology, Karolinska University Hospital, Stockholm, Sweden; 6 Women’s Health and Perinatology Research Group, Department of Clinical Medicine, UiT-The Arctic University of Norway, Tromsø, Norway; 7 Division of Obstetrics and Gynecology, Department of Clinical Science Intervention and Technology (CLINTEC), Karolinska Institutet, K56 Huddinge, 141 86 Stockholm, Sweden; 8 Collage for Physicians and Surgeons, Center for Psychoanalytic Training and Research, Columbia University, New York, USA

**Keywords:** COVID-19, Pregnancy, Perinatal care, Mental health, Depression, Anxiety, Stress

## Abstract

**Background:**

The COVID-19 pandemic has contributed to unprecedented worries and challenges for pregnant women due to social restrictions and changes in maternity care provision. We aimed to investigate the mental health impact of COVID-19 pandemic on pregnant women in Sweden and explore factors associated with poor perinatal mental health in this specific context.

**Method:**

This was a nation-wide cross-sectional survey of pregnant women living in Sweden. Validated questionnaires were distributed through non-profit organizations´ websites and social media channels from May 2020 to February 2021. Perinatal depression, anxiety, and acute stress reaction were assessed using the Edinburgh Postnatal Depression Scale (EPDS), Generalized Anxiety Disorder-7 (GAD-7) and Impact Event Scale (Revised) (IES-R), respectively. Sociodemographic characteristics and self-perceived mental well-being were also obtained. Factors associated with mental health outcomes were analyzed using multivariate logistic regression model.

**Results:**

Among a total of 470 participants, 43.2% (*n* = 203) reported depression (EPDS ≥13**)**, 25.7% (*n* = 121) moderate to severe anxiety (GAD-7 score ≥ 10), and 23.7% (*n* = 110) moderate to severe acute stress reaction (IES-R ≥ 33). 27.4% participants (*n* = 129) expressed concerns regarding their mental well-being during the pandemic. Pregnant mothers who had sick family members reported poorer mental health outcomes than those who did not (median [Interquartile range (IQR)] EPDS scores: 14.0 [8.75–18.0] vs 11.0 [6.25–15.0], *p* < .001; median (IQR) GAD7 scores: 7.0 [4.0–12.25] vs 6.0 [3.0–9.0], *p* = .003); median (IQR) IES-R scores: 20.0 [9.0–38.0] vs 15.0 [7.0–30.0], *p* = .048). Logistic regression analyses revealed that risk factors for poor mental health outcomes were having a sick family member with any illness, unemployment, and experiencing a substantially stressful life event. Having a higher educational level and a younger age during the pandemic were protective.

**Conclusion:**

Depression and anxiety were highly prevalent among pregnant women in Sweden during the COVID-19 pandemic, indicating a need for professional mental health support for this vulnerable group of population. Unemployment was an associated risk factor whereas younger age and higher educational level were protective suggesting an important role of socio-economic factors in modulating the impact of COVID-19 pandemic on perinatal mental health.

**Supplementary Information:**

The online version contains supplementary material available at 10.1186/s12884-022-04553-x.

## Background

The consequences of the COVID-19 pandemic on global mental health are significant and its long-term impact on the global burden of disease is likely to be high. The COVID-19 pandemic has a profound impact on healthcare systems and potentially on pregnancy outcomes [1, 2]. Rapid changes to the delivery of maternity health care services have occurred in many countries across the globe in response to the COVID-19 pandemic. Maternity care provisions are facing a challenge in their attempt to balance the needs and safety of pregnant women and their care providers [3].

Globally, maternal and perinatal outcomes have worsened during the COVID-19 pandemic, with an increase in maternal deaths, stillbirths, ruptured ectopic pregnancies, and maternal depression [4]. During a situation of acute crisis, such as the COVID-19 pandemic, pregnant women are even more vulnerable than the general population in terms of susceptibility to mental health disorders [5]. Prenatal distress and psychiatric symptomatology have also been shown to be more prevalent among pregnant women during the COVID-19 pandemic compared to the pre-pandemic period [6]. Increased vulnerability to mental health disorders among pregnant women could be exaggerated by concerns about potential adverse effect of the COVID-19 infection on the wellbeing of the unborn child as well as the feeling of lack of control over own health due to changes in routine maternity care provisions and service delivery (1). Furthermore, imposed social restrictions might lead to feelings of loneliness, lack of support and insecurity causing more anxiety.

Mental disorders are a common cause of morbidity during pregnancy both in high and low resource countries. Sweden is an affluent country with a well-functioning national healthcare system. In 2020, the total population of Sweden was 10,379,295, the fertility rate was 1.67 children per woman and there were 113,077 total livebirths [7]). In Sweden, the pre-pandemic prevalence of perinatal depression was 13.7% (women with high EPDS scores, i.e. ≥ 12) [8, 9]. that of anxiety symptoms in early pregnancy was 15.6% (HADS-A scores ≥8) [10]. Approximately 12.0% of pregnant women seem to report symptoms of posttraumatic stress in Sweden (assessed by a 3-item questionnaire), although this was not based on a diagnostic questionnaire or a clinical diagnosis [11]. The estimated lifetime prevalence of post-traumatic stress disorder (PTSD) among the general female population in Sweden is 7.4% [12].

Sweden chose a different approach in mitigating the impact of the COVID-19 pandemic, compared to other neighboring Scandinavian countries [13]. Society remained open, while masks were not recommended until the end of 2020. Pregnant women were not considered a vulnerable group initially and until recently they were excluded from being vaccinated according to recommendations of the Swedish National Board of Health and Welfare [14, 15]. During the pandemic, some changes in antenatal and intrapartum care routines occurred that might have been perceived by the pregnant women and their families as less welcoming. The husbands/partners of pregnant women have not been welcomed at the antenatal visits. In some countries, including Sweden, partners have not been allowed to attend the delivery if being tested positive for COVID-19, and no husband/partner has been allowed to stay overnight in the hospital with the woman and the baby during postpartum period.

We hypothesized that the changes and adjustments required in pregnant women’s personal life as well as in the routine maternity care services due to COVID-19 pandemic might have a negative impact on maternal mental health. In this context, this study aimed to investigate the impact of the COVID-19 pandemic on the mental wellbeing of pregnant women in Sweden and explore both traditional (socio-economic factors, life events etc.) and pandemic-specific risk factors associated with poor perinatal mental health.

## Method

### Study design and setting

This study is part of a global survey investigating the impact of the COVID-19 pandemic on pregnant women’s mental health, including perinatal depression, anxiety, and acute stress reaction. This multi-national project includes an anonymous web-based survey distributed to pregnant women in China (Hong Kong SAR and Shanghai), Norway, Sweden, Switzerland, Taiwan and all states of the United States of America (USA). The Swedish part of the survey was started in May 2020 and completed in February 2021.

Pregnant women who 1) resided in Sweden during the COVID-19 pandemic and 2) could understand Swedish could participate in the study. Exclusion criteria were 1) unconfirmed pregnancy status (if the pregnancy was not confirmed by a midwife or a doctor yet) and 2) inability to use a web-based application to answer the survey questionnaires. The pregnant women are recruited via non-profit organizations´ websites and social media channels. Via the web link, the participants were guided to the research website where they could read information about the project and answer the questionnaires anonymously if agreed to participate in the study. Data were collected using Qualtrics, a widely used online survey platform for social science research.

### Measurements

Sociodemographic data were self-reported by the participants, including age, marital status (single, married, cohabitating, in a relationship, divorced), working hours (< 40 h per week, 40 h, > 40 h per week), monthly family income (< 40,000 SEK, 40000 SEK, > 40,000 SEK), and education level (no education, 9-year mandatory education, high school, professional education, bachelor, masters, doctoral). Family health status was assessed by the question, ‘in the past 12 months, do you have any sick family members?’ In addition, the anonymous survey included assessments of symptoms of perinatal depression, anxiety and PTSD using validated instruments [16–18] .

#### Edinburgh postnatal depression scale (EPDS)

Depressive symptoms among pregnant mothers were assessed by EPDS [19]. EPDS is a 10-item self-report scale assessing postnatal depression. Each item offers four options which are scored from 0 to 3, higher indicates more intense depressive symptoms over the past 7 days. Total score of EPDS ranges from 0 to 30. The Swedish version of Edinburgh Postnatal Scale is well validated [16] and commonly used in women mental health studies, with an optimal cut-off score of ≥13. In our study, the scale demonstrated good internal consistency with a Cronbach’s Alpha of .87.

#### Generalized anxiety Disorder-7 (GAD7)

The GAD7 is a globally used self-report scale that assesses participants’ level of generalized anxiety in the past 14 days. The 7-item Likert-4 scale measures the frequency of being distressed by anxiety-related symptoms, with options ranging from *not at all* to *nearly every day* (scored from 0 to 3). Total score of GAD7 ranges from 0 to 21, higher scores indicate a higher level of generalized anxiety. The total score of GAD7 will be further categorized into severity categories of minimal (0–4), mild (5–9), moderate (10–14), and severe (15–21) anxiety. In this study, we used the Swedish version of GAD7 [20], which has demonstrated excellent internal consistency in previous studies.

#### Impact event scale (revised) (IES-R)

The IES-R is a 22 item self-reported measure designed to assess acute stress reactions and probable post-traumatic distress following stressful life events in the past 7 days [21]. Each item measures with a Likert-5 rating scale (from *not at all* to *extremely,* 0–4). Total score of IES-R ranges from 0 to 88, higher scores indicate higher levels of distress from acute stress reactions. The total scores are further categorized into categories of minimal (0–23), mild (24–32), moderate to severe concern for PTSD (33–88). In this study, IES-R is administered as event-oriented to the covid-19 pandemic. We used the Swedish version of IES-R [18, 22], which has demonstrated an excellent internal consistency with a Cronbach’s Alpha of .94 in this study.

#### Mental wellbeing and stressful life events during the pandemic

The perceived mental well-being of participants was assessed by a single 10-point Likert scale enquiring overall mental health status in the past 12 months. Participants were then asked to fill in a stressful event checklist assessing their exposures in the past 1 year, which included marriage, divorce, death in the family, career change, unemployment, and other.

### Statistical analysis

Participants’ characteristics and scores obtained from mental health assessments were analyzed by descriptive statistics. Continuous variables are presented as means and standard deviations (SD), while categorical variables are presented as number (n) and percentage (%). Associations were analyzed using Chi-squared test. The total scores of the three mental health status measurement tools (EPDS, GAD7 and IES-R) were not normally distributed, and so are presented as medians with interquartile ranges (IQR). Non-parametric Mann-Whitney U test was applied to compare the severity of symptoms between families with sick family members (including COVID-19 and other illnesses) and those without sick family members. Associations between mental health outcomes (depression and anxiety, acute stress reactions) and demographic characteristics of participants were estimated by univariate and multivariate logistic regression, and are presented as odds ratios (ORs) with 95% Confidence intervals (CIs). The statistical significance was set at a *p-*value of < 0.05. All statistical analyses were performed with the Statistical Package for the Social Science (SPSS) version 26 (IBM® SPSS® Statistics).

## Results

### Demographic characteristics of the participants

Of a total of 522 study participants, 52 women were excluded as their responses indicated that they had either given birth or were not pregnant at the time when they replied to the survey questionnaires. Thus, data from 470 pregnant women who completed the survey were analyzed. The sociodemographic characteristics of the participants, their self-perceived overall mental health status, and stressful event exposure are presented in Table 1. The mean age of the participants was 30.72 (*SD* = 4.21) years. Most women lived in cohabitation with a partner (*n* = 259, 55.1%) or were married (*n* = 195, 41.5%). The majority (*n* = 271, 57.7%) were working 40 h a week. Two third of the participants (*n* = 283, 60.2%) had a bachelor degree or above, and 30.2% (*n* = 142) had at least one sick family member.

**Table 1:** Sociodemographic characteristics, self-perceived overall mental health status, and stressful event exposure reported by the participants (*N* = 470)

		n, %			
			Family Health Status		
**Variables**	***M, SD***	**Total**	**Families with sick members**	**Families without sick members**	***p***
**Overall**		470 (100.0)			
**Age**	30.72 (4.21)				
18–25		50 (10.6)	17 (12.0)	33 (10.1)	.740
26–30		180 (38.3)	57 (40.1)	123 (37.5)	
31–40		232 (49.4)	65 (45.8)	167 (50.9)	
> 40		8 (1.7)	3 (2.1)	5 (1.5)	
**Civil Status**					
Single		3 (0.6)	1 (0.7)	2 (0.6)	.661
Married		195 (41.5)	59 (41.5)	136 (41.5)	
Cohabitating		259 (55.1)	77 (54.2)	182 (55.5)	
in a relationship		12 (2.6)	4 (2.8)	8 (2.4)	
Divorced		1 (0.2)	1 (0.7)	0 (0)	
**Working Hours**					
< 40 h per week		156 (33.2)	50 (35.2)	106 (32.3)	.241
40 h per week		271 (57.7)	75 (52.8)	196 (59.8)	
> 40 h per week		43 (9.1)	17 (12.0)	26 (7.9)	
**Monthly Family Income**					
< 40,000 SEK		159 (33.8)	38 (26.8)	121 (36.9)	.052
40,000 SEK		198 (42.1)	70 (49.3)	128 (39.0)	
> 40,000 SEK		103 (21.9)	28 (19.7)	75 (22.9)	
**Educational Level**					
No education		2 (0.4)	0 (0)	2 (0.6)	.896
9-year mandatory education		10 (2.1)	4 (2.8)	6 (1.8)	
High school		96 (20.4)	29 (20.4)	67 (20.4)	
Professional education		79 (16.8)	26 (18.3)	53 (16.2)	
Bachelor		146 (31.1)	40 (28.2)	106 (32.3)	
Masters		127 (27.0)	40 (28.2)	87 (26.5)	
Doctoral		10 (2.1)	3 (2.1)	7 (2.1)	
**Overall Mental Health Status (1–10)**	4.61 (1.88)				
Healthy		341 (72.6)	89 (62.7)	252 (76.8)	**.002**
Worsened Mental Health (≥6)		129 (27.4)	53 (37.3)	76 (23.2)	
**Stressful event Exposure**					
Giving Birth		54 (11.5)	15 (10.6)	39 (11.9)	.679
Marriage		33 (7.0)	10 (7.0)	23 (7.0)	.991
Divorce		0 (0)	0 (0)	0 (0)	N/A
Death in families		54 (11.5)	20 (14.1)	34 (10.4)	.246
Changing Career		70 (14.9)	19 (13.4)	51 (15.5)	.544
Exams		27 (5.7)	8 (5.6)	19 (5.8)	.946
Unemployment		35 (7.4)	12 (8.5)	23 (7.0)	.585
Others		131 (27.9)	31 (21.8)	100 (30.5)	.055
**No. of stressful event exposure**					
0		181 (38.5)	58 (40.8)	123 (37.5)	.409
1		195 (41.5)	60 (42.3)	135 (41.2)	
2		74 (15.7)	18 (12.7)	56 (17.1)	
3		19 (4.0)	5 (3.5)	14 (4.3)	
4		1 (0.2)	1 (0.7)	0 (0)	

### Mental health status of the pregnant mothers and severity categories

The overall prevalence of probable depression for pregnant women during the pandemic (EPDS ≥13) was 43.2% (*n* = 203). One fourth (*n* = 121, 25.7%) of Swedish pregnant women participating in the study displayed moderate to severe generalized anxiety symptoms (GAD-7 ≥ 10). 23.7% (*n* = 110) of the participants had moderate to severe concern for PTSD (IES-R ≥ 33).

Women whose families had sick members with any illness in the past 12 months reported experiencing more depression, anxiety (probable depression: *n* = 81, 57.0% vs *n =* 122, 37.2%, *p* < .001; moderate to severe anxiety: *n* = 50, 35.2% vs *n* = 71, 21.6%, *p* = .015) but not acute stress reactions (moderate to severe concern for probable PTSD: *n =* 38, 27.1% vs *n* = 72, 22.2%, *p* = .503(Table 2).

**Table 2:** Self-reported mental health status and severity of depression, anxiety, and acute stress in total study cohort and subgroups

Severity Category	N, %	***M*** (SD)	Families with sick members		Families without sick members		***p***
			N, %	***M*** (SD)	N, %	***M*** (SD)	
**EPDS, depression symptoms**							
Total cohort	470 (100)	11.76 (6.23)	142 (100)	13.39 (6.52)	328 (100)	11.05 (5.97)	<.001
Normal	267 (56.8)		61 (43.0)		206 (62.8)		
Probable Depression (scores ≥13)	203 (43.2)		81 (57.0)		122 (37.2)		
**GAD7, Anxiety**							
Total cohort	470 (100)	7.00 (5.25)	142 (100)	8.09 (5.58)	328 (100)	6.53 (5.04)	.015
Normal (0–4)	175 (37.2)		45 (31.7)		130 (39.6)		
Mild (5–9)	174 (37.0)		47 (33.1)		127 (38.7)		
Moderate (10–14)	66 (14.0)		25 (17.6)		41 (12.5)		
Severe (15–21)	55 (11.7)		25 (17.6)		30 (9.1)		
**IES-R, Acute Stress symptoms**							
Total cohort	470 (100)	21.16 (17.49)	140 (100)	23.60 (19.05)	328 (100)	20.11 (16.69)	.503
Normal (0–23)	308 (65.5)		88 (62.0)		220 (67.1)		
Mild (24–32)	46 (9.8)		14 (9.9)		32 (9.8)		
Moderate to Severe concern for PTSD (33–88)	110 (23.7)		38 (27.1)		72 (22.2)		

*Abbreviations*: *EPDS* 10-item Edinburgh Postnatal Depression Scale, *GAD-7* 7-item Generalized Anxiety Disorder, *IES-R* 22-item Impact of Event Scale–Revised

#### Scores of measurements

The total median scores (IQR) on the EPDS for depression, GAD7 for anxiety, and IES-R for acute stress reaction among women were 11.0 (7.0–16.0), 6.0 (3.0–10.0), and 16.0 (7.0–31.75) respectively. Similar to findings in severity categories of women, women whose families had sick members had higher scores for all mental heatlh outcomes compared with those who did not (median EPDS scores: 14.0 (8.75–18.0) vs 11.0 (6.25–15.0), *p* < .001; median GAD7 scores: 7.0 (4.0–12.00) vs 6.0 (3.0–9.0), *p* = .003; median IES-R scores: 18.5 (7.25–34.0) vs 15.0 (7.0–30.0), *p* = .048) (Table 3).

**Table 3:** Scores of Mental health Measurements in Total Cohort and Subgroups

Scale	Total score, Median	IQR	Families with sick members		Families without sick members		***P***
			Total score, Median	IQR	Total score, Median	IQR	
**EPDS, Depression Symptoms**	11.0	7.0–16.0	14.0	8.00–18.0	11.0	6.25–15.0	<.001
**GAD7, Anxiety Symptoms**	6.0	3.0–10.0	7.0	4.0–12.00	6.0	3.0–9.0	.003
**IES-R, Acute Stress Reaction**	16.0	7.0–31.75	18.5	7.25–34.0	15.0	7.0–30.0	.048

*Abbreviations*: *EPDS* 10-item Edinburgh Postnatal Depression Scale, *GAD-7* 7-item Generalized Anxiety Disorder, *IES-R* 22-item Impact of Event Scale–Revised, *IQR* Interquartile Range

#### Factors associated with mental health status of pregnant women

Multivariable logistic regression analyses were performed to investigate factors associated with the mental health status of pregnant women during the pandemic. Having sick family members in the past 12 months increased the odds of depression by 2.6 times (aOR 2.627, 95% CI [1.686, 4.092], *p* < .001), anxiety by 2.2 times (aOR 2.218, 95% CI [1.376, 3.573] *p* = .001), and perceived worsening mental health in the past 12 months by 2.2 times (aOR 2.246, 95% CI [1.409, 3.580], *p* = .001). Birth giving increased the odds of acute stress reaction by 2.4 times (aOR 2.407, 95% CI [1.260, 4.597], *p* = .008). Unemployment increased the odds of depression by 2.7 times (aOR 2.759, 95% CI [1.180, 6.453], *p* = .019) and acute stress reaction by 3.2 times (aOR 3.242, 95% CI [1.476, 7.120], *p* = .003). Encountering an exceptional stressful life event increased the odds of depression (aOR 1.955, 95% CI [1.232, 3.101], *p* = .004), anxiety (aOR 2.427, 95% CI [1.471, 4.005], *p* = .001), and perceived worsening mental health in the past 12 months (aOR 2.160, 95% CI [1.326, 3.520], *p* = .002) (Tables 4, 5, 6, 7).

**Table 4:** Factors associated with depressive symptoms (EPDS ≥13) among pregnant women

	cOR	95% CI	***p***	aOR	95% CI	***p***
Age						
18–25	Ref	Ref		Ref	Ref	
26–30	.394	.205–.759	**.005**	.490	.233–1.027	.059
31–40	.321	.169–.609	**.001**	.424	.196–.916	**.029**
> 40	.309	.066–1.451	.137	.371	.068–2.016	.251
Working Hours						
< 40 h per week	Ref	Ref		Ref	Ref	
40 h per week	.633	.425–.942	**.024**	.793	.503–1.251	.319
> 40 h per week	.870	.442–1.710	.686	1.044	.487–2.240	.912
Monthly Family Income						
< 40,000 SEK	Ref	Ref		Ref	Ref	
40,000 SEK	1.210	.795–1.841	.375	.785	.475–1.296	.344
> 40,000 SEK	.750	.450–1.250	.270	.865	.498–1.505	.068
Educational Level						
Bachelor or above	.429	.294–.627	**<.001**	.563	.357–.889	**.014**
Family Health Status						
With sick family members	2.242	1.502–3.347	**<.001**	2.627	1.686–4.092	**<.001**
Giving Birth						
Yes	1.364	.773–2.406	.284	1.523	.817–2.839	.185
Marriage						
Yes	1.104	.542–2.247	.786	.920	.416–2.037	.837
Death in families						
Yes	1.254	.710–2.213	.435	1.361	.735–2.522	.327
Changing Career						
Yes	.693	.409–1.174	.173	.809	.444–1.474	.489
Exams						
Yes	.535	.230–1.249	.148	.626	.240–1.631	.338
Unemployment						
Yes	3.610	1.692–7.702	**.001**	2.759	1.180–6.453	**.019**
Others						
Yes	1.703	1.134–2.556	**.010**	1.955	1.232–3.101	**.004**

*p < .05, **p < .01

**Table 5:** Factors associated with anxiety symptoms (GAD7 ≥ 10) among pregnant women

	cOR	95% CI	***p***	aOR	95% CI	***p***
Age						
18–25	Ref	Ref		Ref	Ref	
26–30	.403	.211–.771	**.006**	.404	.193–.845	**.016**
31–40	.331	.175–.625	**.001**	.323	.149–.709	**.005**
> 40	.168	.019–1.466	.106	.214	.022–2.051	.181
Working Hours						
< 40 h per week	Ref	Ref		Ref	Ref	
40 h per week	.903	.575–1.419	.658	.998	.596–1.670	.994
> 40 h per week	1.354	.652–2.812	.416	1.325	.576–3.049	.508
Monthly Family Income						
< 40,000 SEK	Ref	Ref		Ref	Ref	
40,000 SEK	1.379	.843–2.254	.200	.911	.509–1.630	.753
> 40,000 SEK	1.511	.855–2.670	.156	1.778	.966–3.274	.065
Educational Level						
Bachelor or above	.582	.383–.883	**.011**	.691	.413–1.157	.160
Family Health Status						
With sick family members	1.967	1.276–3.304	**.002**	2.218	1.376–3.573	**.001**
Giving Birth						
Yes	1.125	.596–2.123	.717	1.338	.668–2.680	.412
Marriage						
Yes	1.088	.491–2.411	.835	.926	.388–2.212	.863
Death in families						
Yes	1.247	.668–2.329	.488	1.296	.668–2.514	.444
Changing Career						
Yes	1.088	.614–1.930	.772	1.338	.708–2.529	.369
Exams						
Yes	.641	.237–1.731	.380	.944	.323–2.758	.915
Unemployment						
Yes	2.328	1.151–4.708	**.019**	1.798	.812–3.983	.148
Others						
Yes	1.905	1.226–2.961	**.004**	2.427	1.471–4.005	**.001**

*p < .05, **p < .01

**Table 6:** Factors associated with acute stress symptoms (IES-R ≥ 33) among pregnant women

	cOR	95% CI	***p***	aOR	95% CI	***p***
Age						
18–25	Ref	Ref		Ref	Ref	
26–30	.437	.226–.843	**.014**	.634	.301–1.334	.229
31–40	.323	.168–.620	**.001**	.491	.222–1.085	.079
> 40	.829	.178–3.856	.811	1.063	.193–5.854	.944
Working Hours						
< 40 h per week	Ref	Ref		Ref	Ref	
40 h per week	.697	.441–1.102	.697	.973	.577–1.640	.917
> 40 h per week	.807	.365–1.782	.595	1.034	.417–2.564	.943
Monthly Family Income						
< 40,000 SEK	Ref	Ref		Ref	Ref	
40,000 SEK	1.203	.744–1.944	.451	.764	.430–1.356	.358
> 40,000 SEK	.585	.306–1.117	.104	.667	.334–1.332	.252
Educational Level						
Bachelor or above	.397	.257–.615	**<.001**	.541	.321–.913	**.021**
Family Health Status						
With sick family members	1.304	.827–2.056	.253	1.342	.812–2.218	.252
Giving Birth						
Yes	2.295	1.265–4.163	**.006**	2.407	1.260–4.597	**.008**
Marriage						
Yes	.857	.362–2.033	.727	.665	.254–1.742	.406
Death in families						
Yes	.705	.342–1.451	.342	.812	.378–1.741	.592
Changing Career						
Yes	.437	.209–.912	.**027**	.502	.222–1.136	.098
Exams						
Yes	.386	.114–1.306	.126	.612	.169–2.221	.459
Unemployment						
Yes	3.879	1.923–7.825	**<.001**	3.242	1.476–7.120	**.003**
Others						
Yes	1.385	.871–2.203	.168	1.338	.796–2.249	.271

*p < .05, **p < .01

**Table 7:** Factors associated with worsen mental health (Global rating scale ≥6) among pregnant women

	cOR	95% CI	***p***	aOR	95% CI	***p***
Age						
18–25	Ref	Ref		Ref	Ref	
26–30	.439	.230–.838	**.012**	.497	.239–1.034	.061
31–40	.348	.184–.656	**.001**	.391	.180–.852	**.018**
> 40	1.174	.264–5.226	.833	1.447	.283–7.409	.657
Working Hours						
< 40 h per week	Ref	Ref		Ref	Ref	
40 h per week	.611	.394–.947	**.028**	.692	.422–1.134	.144
> 40 h per week	1.220	.604–2.464	.579	1.292	.589–2.833	.523
Monthly Family Income						
< 40,000 SEK	Ref	Ref		Ref	Ref	
40,000 SEK	1.136	.717–1.802	.587	.812	.468–1.408	.458
> 40,000 SEK	.710	.395–1.275	.251	.864	.464–1.609	.645
Educational Level						
Bachelor or above	.573	.380–.863	**.008**	.779	.471–1.289	.332
Family Health Status						
With sick family members	1.975	1.290–3.023	**.002**	2.246	1.409–3.580	**.001**
Giving Birth						
Yes	1.129	.606–2.105	.703	1.206	.615–2.366	.586
Marriage						
Yes	1.799	.867–3.732	.115	1.771	.810–3.876	.152
Death in families						
Yes	1.374	.749–2.519	.304	1.463	.767–2.790	.248
Changing Career						
Yes	.684	.371–1.260	.223	.863	.441–1.690	.667
Exams						
Yes	.585	.217–1.578	.289	.831	.284–2.430	.735
Unemployment						
Yes	1.625	.793–3.331	.185	1.096	.485–2.478	.825
Others						
Yes	1.842	1.193–2.844	**.006**	2.160	1.326–3.520	**.002**

*p < .05,**p < .01

Having a higher level of education (i.e. having a bachelor degree or above) decreased the odds of depression (aOR .563, 95% CI [.357, .889], *p* = .014) and acute stress reaction (aOR .541, 95% CI [.321, .913], *p* = .021). Women in the age group of 26 to 30 were less likely to develop both depression (aOR .424, 95% CI [.196, .916], *p* = .029) and anxiety (aOR .323, 95% CI [.149. .709], *p* = .005), and less likely to have perceived worsening mental health in the past 12 months (aOR .391, 95% CI [.180, .852], *p* = .018). Women in the age group of 18 to 25 were less likely to develop anxiety (aOR .404, 95% CI [.193, .845], *p* = .016) (Tables 4, 5, 6, 7).

#### Supplementary analyses on multiple event exposure

To investigate the effect of multiple event exposure, the total number of stressful event exposures was entered into the multivariable logistic regression models, replacing the single event exposure variables. After controlling all other variables, Increase in the number of stressful encounter increases the odds of depression and anxiety (depression: aOR 1.311, 95% CI [1.036, 1.659], *p* = .024; anxiety: aOR 1.445, 95% CI [1.119, 1.865], *p* < .005), but not acute stress reaction (aOR 1.159, 95% CI [.894, 1.501], *p* = .266) (Supplementary Tables 1, 2, 3).

## Discussion

Women’s healthcare is often adversely affected by humanitarian disasters [23]. Our findings highlight the importance of having robust maternity services able to cope and provide adequate care and support to women in such emergency situations, in line with the findings of a recent review on the impact of the COVID-19 pandemic on pregnant women [4]. Pregnant women who participated in our study reported a high prevalence of symptoms of perinatal depression, anxiety, and acute stress reaction. One of the first studies conducted under the COVID-19 pandemic in China showed that pregnant women had significantly higher rates of depressive symptoms (26.0% vs 29.6%, *P* = 0.02) than women assessed before the pandemic was declared [24]. Similar findings have been reported also from affluent Western countries, such as Canada [6] and in a scoping review conducted to compile evidence on direct and indirect impacts of the pandemic on maternal health [25].

In our study, the prevalence of depressive symptoms among pregnant women in Sweden was exceptionally high, both in comparison with pre-pandemic prevalence studies from Sweden [9], neighboring Scandinavian countries [9], as well as internationally [26, 27]. This result stands in contrast to the expectations that in an open, non-confined society, such as Sweden, pregnant women would have better mental well-being compared to societies with prolonged isolation during the pandemic.

The inclination of “adopting social distancing measures” is often thought to be positively associated with worsened mental health during the pandemic. However, it has proven not to be the case in a study in New York City [28]. Silverman et al. found that in women with low socioeconomic status, who are most vulnerable for prenatal mood disruption, the social restrictions reduced their mental health problems. A decrease in symptomatology was also found in a recent Gallup Panel 2021 [29] reporting a decrease in worry after restrictions were put in place, compared to earlier in the pandemic. Therefore, loose social restrictions leading to worries for disease transmission could be a potential explanation of why the Swedish pregnant reported high prevalence of mental health problems.

The prevalence of depression (EPDS scores of ≥13) among Swedish pregnant women during the pandemic was high at 42.5% compared to 12% before the pandemic [30], and the mean EPDS score had doubled (mean: 5.0, *n* = 110 vs mean: 11.63, *n* = 470). Similarly, the pre-pandemic prevalence of anxiety symptoms in pregnant women residing in Sweden (HADS-A scores ≥8 during early pregnancy) was 15.6% (Rubertsson et al., 2014), while during the pandemic the point prevalence (GAD7 scores ≥10) has increased to 25.3%. However, this needs to be interpreted with caution as the women participating in our study were at variable stages in their pregnancy.

With regards to acute stress reaction, the estimated lifetime prevalence of acute stress and in severe conditions of probable PTSD among women in Sweden is 7.4% [31] using diagnostic procedures and the PTSD Checklist (PCL), a series of posttraumatic stress scales to assess acute stress and PTSD developed by Persson [12]. Current PTSD, based on diagnostic criteria and severity, was reported by 4.1% (95% CI 2.8–5.8) of the pregnant women [12]. Persson et al. found in their study conducted in 2017–2018, that a majority of pregnant women with PTSD experienced violence, and expressed a fear of childbirth (Persson et al., 2020). Both domestic violence and fear of childbirth have increased globally, as a consequence of the COVID-19 pandemic [6]. Although we did not inquire about domestic violence in our survey, these results are important to consider, in relation to the present study. In our study, the prevalence of moderate to severe concern for probable PTSD among the Swedish pregnant women during the pandemic was 23.4%. The rise in acute stress reaction and PTSD symptoms has also been shown in previous research during the SARS and MERS outbreaks (Lee et al., 2007; Lee et al., 2019; Lee et al., 2018; Mak et al., 2010; Park et al., 2020). Similar results occurred after the 9/11 terrorist attack [32] and the Holocaust (Bowers & Yehuda, 2016; Yehuda et al., 2005; Yehuda et al., 1998). The clinically concerning acute stress in pregnant women may impact their fetuses (Doyle et al., 2015). Chronic stress may cause an epigenetic change in the placenta, facilitating cortisol to pass through easier, which can result in the fetus brain maturing faster, and upon birth being hypervigilant (Monk, 2016; Monk et al., 2016; O’Connor et al., 2016). Beside the potential impact on the fetus, there is a risk of increase in number of women experiencing fear of labor and childbirth, which is important for healthcare professionals to be aware of to avoid unnecessary operative deliveries and associated complications.

When we compared participants, who were from families that had sick members and those who were not, we found that poor family health status had increased the odds of having perinatal mental health issues, including poorer outcomes in depression and anxiety. Pregnant women who had sick family members were more likely to score over the cutoff of EPDS and GAD7. This is a good indication and stratifying strategy, potentially applied in early mental health prevention. Since family health status traditionally being considered a precipitating and maintaining factor for health anxiety in family medicine.

Unemployment and exceptionally stressful encounters were also associated with an increasing likelihood of exhibiting probable perinatal mental disorders during the COVID-19 pandemic. This is not surprising as lower socio-economic status is known to be associated with higher risk of perinatal mental health problems. Rectifying long-standing health inequalities in the societies is equally important while making swift responses to the impact of the pandemic.

In our study, women in the age of 26 to 40 years showed significantly lower levels of anxiety. It could be related to the fact that in this age group, most mothers have stable families, economic stability, and good social networks.

### Strengths and limitations

Our study is one of the first in Sweden to evaluate mental health of pregnant women during COVID-19 pandemic. Data collection took place form May 2020 to February 2021, initiating the data collection during the peak of the pandemic in Sweden. Due to the long timeframe, both peaks and lows with regards to the severity of the pandemic were included. However, our study is not without limitations. Convenient sampling method used limits the generalizability of our findings, and the cross-sectional design did not allow to explore the trends over a longer time and differences in mental health status of pregnant women during different stages of gestation and during different severities of the pandemic. The study population of 470 pregnant women, represents only a tiny fraction of women who were pregnant during the study period as the total number of livebirths during this period was 93,528. Furthermore, as the questionnaires had to be filled out online without having any direct contact with the researchers and inability to use a web-based application to answer the survey questionnaires was one of the exclusion criteria, women who were not able to read or did not have excess to internet could not participate in this survey introducing sampling bias/selection bias. Moreover, misinterpretation of questions by participants cannot be excluded as the survey was self-administered online. The prevalence of perinatal mental health disorders can be affected by pre-existing comorbidities. Although, we did not ask specific questions about pre-existing physical or mental illness, our question about overall mental health status should have captured any history of psychiatric morbidity.

The prevalence of anxiety, depression, and stress in pregnant women was found to be very high, and future studies with larger sample size are needed to evaluate the prevalence of perinatal mental health disorders due to the COVID-19 pandemic in Sweden. However, a much larger survey performed in the last week of April 2021 in neighboring Norway has shown similar results with 32% (1164/3642) of women having postpartum depressive symptoms compared to 10% (225/2217) in a pre-pandemic survey [33]. Generally, the stressful life events refer to experiences in the past 1 year. Therefore, in a small number of women who participated in the survey early on and were in first trimester of pregnancy, these events might partly relate to events occurring before the COVID-19 pandemic. However, this is unlikely to have significantly affected our findings.

The consequences on global mental health due to COVID-19 will be significant and cause long-term impact on the global burden of disease. Mental health problems cannot not be overlooked in this regard. It is important to understand what women need to cope with and navigate their pregnancies during this pandemic. Since pregnant women reported frequently reading and searching for information on social media, health care professionals have to become more engaged in informing pregnant women via different channels to ensure accuracy of information accessible to them. It is important that women receive reliable information and advice from their healthcare providers rather than from the social media as a primary source of information.

The high prevalence of perinatal mental health problems found in this study suggests the need for a systematic approach to screening pregnant women and providing professional support to those at high risk. Providing psychological first aid and counselling are essential during a pandemic. It helps in reducing the psychological distress and promoting adaptive coping strategies to deal with the situation short and long term [34, 35]. Web-based psychosocial support can be an invaluable resource to bridge the treatment gap of perinatal mental health problems (Schwank et al., 2020), which has clearly increased during the global pandemic. Results from pre-pandemic research on the effect of internet-based support for perinatal mental health care services in Sweden, have shown significantly lower levels of depressive symptoms post treatment. Pregnancy adapted internet based psychosocial support for antenatal depression is feasible, acceptable and efficacious [36] and could be especially suitable in a pandemic.

## Conclusion

Depression and anxiety were highly prevalent among pregnant women in Sweden during the COVID-19 pandemic indicating a need for professional mental health support for this vulnerable group of population. Unemployment was an associated risk factor whereas younger age and higher educational level were protective suggesting an important role of socio-economic factors in modulating the impact of COVID-19 pandemic on perinatal mental health.

## Supplementary Information

**Additional file 1: Table S1.** Factors associated with depressive symptoms (EPDS ≥13) among pregnant women. **Table S2.** Factors associated with anxiety symptoms (GAD7 ≥ 10) among pregnant women. **Table S3. **Factors associated with acute stress symptoms (IES-R ≥ 33) among pregnant women.

## Data Availability

The dataset generated and analyzed in the current study is not publicly available. However, anonymized data can be made available from the corresponding author on reasonable request. The data was collected with informed consent, provided by the participants on the specific research project on perinatal mental health during the Covid-19 pandemic. Therefore, cannot be made available publicly.

## Abbreviations

aOR: Adjusted Odds Ratio
CI: Confidence Interval
cOR: Crude Odds Ratio
COVID-19: Coronavirus Disease 2019
EPDS: Edinburgh Postnatal Depression Scale
GAD7: Generalized Anxiety Disorder 7
HADS-A: Hospital Anxiety and Depression Scale – Anxiety
IES-R: Impact of Event Scale -Revised
IQR: Interquartile Range
MERS: Middle East respiratory syndrome
PTSD: Post-traumatic stress disorder
SARS: Severe acute respiratory syndrome
SEK: Swedish Krona
