# Supplementary material for: Self-reported mental health status of pregnant women in Sweden during the COVID-19 pandemic: a cross-sectional survey
Source: BMC Pregnancy Childbirth. 2022 Mar 28;22:260. doi: 10.1186/s12884-022-04553-x (PMC8960205; doi:10.1186/s12884-022-04553-x)
Supplement: Supplementary file 1 — Additional file 1: Table S1. Factors associated with depressive symptoms (EPDS ≥13) among pregnant women. Table S2. Factors associated with anxiety symptoms (GAD7 ≥ 10) among pregnant women. Table S3. Factors associated with acute stress symptoms (IES-R ≥ 33) among pregnant women. [file 12884_2022_4553_MOESM1_ESM.docx]

| **Supplementary Table. 1**  *Factors associated with depressive symptoms (EPDS ≥ 13) among pregnant women* | | | | | | |
| --- | --- | --- | --- | --- | --- | --- |
|  | **cOR** | **95% CI** | ***p*** | **aOR** | **95% CI** | ***p*** |
| Age |  |  |  |  |  |  |
| 18-25 | Ref | Ref |  | Ref | Ref |  |
| 26-30 | .437 | .226 - .843 | **.014** | .448 | .217 - .925 | **.030** |
| 31-40 | .323 | .168 - .620 | **.001** | .424 | .199 - .904 | **.026** |
| >40 | .829 | .178 – 3.856 | .811 | .377 | .071 – 2.006 | .377 |
| Working Hours |  |  |  |  |  |  |
| <40 hours per week | Ref | Ref |  | Ref | Ref |  |
| 40 hours per week | .633 | .425 - .942 | **.024** | .771 | .497 – 1.197 | .247 |
| >40 hours per week | .870 | .442 – 1.710 | .686 | .971 | .460 – 2.050 | .938 |
| Monthly Family Income |  |  |  |  |  |  |
| <40000 SEK | Ref | Ref |  | Ref | Ref |  |
| 40000 SEK | 1.210 | .795 – 1.841 | .375 | .780 | .481 – 1.265 | .314 |
| >40000 SEK | .750 | .450 – 1.250 | .270 | .794 | .461 – 1.367 | .405 |
| Educational Level |  |  |  |  |  |  |
| Bachelor or above | .429 | .294 - .627 | **<.001** | .503 | .323 - .782 | **.002** |
| Family Health Status |  |  |  |  |  |  |
| With sick family members | 2.242 | 1.502 – 3.347 | **<.001** | 2.482 | 1.615 – 3.816 | **<.001** |
| Event Exposure |  |  |  |  |  |  |
| Total number of stressful event exposure | 1.288 | 1.034 – 1.603 | **.024** | 1.311 | 1.036 – 1.659 | **.024** |
| ****p* <.05, ***p* <.01** | | | | | | |

| **Supplementary Table. 2**  *Factors associated with anxiety symptoms (GAD7 ≥ 10) among pregnant women* | | | | | | |
| --- | --- | --- | --- | --- | --- | --- |
|  | **cOR** | **95% CI** | ***p*** | **aOR** | **95% CI** | ***p*** |
| Age |  |  |  |  |  |  |
| 18-25 | Ref | Ref |  | Ref | Ref |  |
| 26-30 | .403 | .211 - .771 | **.006** | .393 | .191 - .808 | **.011** |
| 31-40 | .331 | .175 - .625 | **.001** | .338 | .156 - .732 | **.006** |
| >40 | .168 | .019 – 1.466 | .106 | .205 | .022 – 1.922 | .165 |
| Working Hours |  |  |  |  |  |  |
| <40 hours per week | Ref | Ref |  | Ref | Ref |  |
| 40 hours per week | .903 | .575 – 1.419 | .658 | .969 | .590 – 1.594 | .902 |
| >40 hours per week | 1.354 | .652 – 2.812 | .416 | 1.311 | .582 – 2.957 | .514 |
| Monthly Family Income |  |  |  |  |  |  |
| <40000 SEK | Ref | Ref |  | Ref | Ref |  |
| 40000 SEK | 1.379 | .843 – 2.254 | .200 | .879 | .499 – 1.550 | .657 |
| >40000 SEK | 1.511 | .855 – 2.670 | .156 | 1.625 | .892 – 2.961 | .113 |
| Educational Level |  |  |  |  |  |  |
| Bachelor or above | .582 | .383 - .883 | **.011** | .658 | .400 – 1.083 | .100 |
| Family Health Status |  |  |  |  |  |  |
| With sick family members | 1.967 | 1.276 – 3.304 | **.002** | 2.065 | 1.300 – 3.281 | **.002** |
| Event Exposure |  |  |  |  |  |  |
| Total number of stressful event exposure | 1.379 | 1.084 – 1.754 | **.009** | 1.445 | 1.119 – 1.865 | **.005** |
| ****p* <.05, ***p* <.01** | | | | | | |

| **Supplementary Table. 3**  *Factors associated with acute stress symptoms (IES-R ≥ 33) among pregnant women* | | | | | | |
| --- | --- | --- | --- | --- | --- | --- |
|  | **cOR** | **95% CI** | ***p*** | **aOR** | **95% CI** | ***p*** |
| Age |  |  |  |  |  |  |
| 18-25 | Ref | Ref |  | Ref | Ref |  |
| 26-30 | .437 | .226 - .843 | **.014** | .564 | .277 – 1.148 | .114 |
| 31-40 | .323 | .168 - .620 | **.001** | .481 | .224 – 1.030 | .059 |
| >40 | .829 | .178 – 3.856 | .811 | 1.178 | .227 – 6.103 | .845 |
| Working Hours |  |  |  |  |  |  |
| <40 hours per week | Ref | Ref |  | Ref | Ref |  |
| 40 hours per week | .697 | .441 – 1.102 | .697 | .896 | .547 – 1.469 | .665 |
| >40 hours per week | .807 | .365 – 1.782 | .595 | .843 | .352 – 2.020 | .702 |
| Monthly Family Income |  |  |  |  |  |  |
| <40000 SEK | Ref | Ref |  | Ref | Ref |  |
| 40000 SEK | 1.203 | .744 – 1.944 | .451 | .814 | .470 – 1.408 | .461 |
| >40000 SEK | .585 | .306 – 1.117 | .104 | .612 | .313 – 1.197 | .151 |
| Educational Level |  |  |  |  |  |  |
| Bachelor or above | .397 | .257 - .615 | **<.001** | .465 | .283 - .765 | **.003** |
| Family Health Status |  |  |  |  |  |  |
| With sick family members | 1.304 | .827 – 2.056 | .253 | 1.295 | .799 – 2.100 | .294 |
| Event Exposure |  |  |  |  |  |  |
| Total number of stressful event exposure | 1.163 | .906 – 1.491 | .235 | 1.159 | .894 – 1.501 | .266 |
| ****p* <.05, ***p* <.01** | | | | | | |
